# Early prediction of acute respiratory distress syndrome complicated by acute pancreatitis based on four machine learning models

**DOI:** 10.1016/j.clinsp.2023.100215

**Published:** 2023-05-03

**Authors:** Mengran Zhang, Mingge Pang

**Affiliations:** a Gastroenterology Department, Xuanwu Hospital Capital Medical University, Beijing, China; b Internal Medicine Department, Beijing Puren Hospital, Beijing, China

**Keywords:** Acute respiratory distress syndrome, Acute pancreatitis, Machine learning, Prediction model

## Abstract

**Background:**

Acute Respiratory Distress syndrome (ARDS) is a common complication of Acute Pancreatitis (AP) and is associated with high mortality. This study used Machine Learning (ML) to predict ARDS in patients with AP at admission.

**Methods:**

The authors retrospectively analyzed the data from patients with AP from January 2017 to August 2022. Clinical and laboratory parameters with significant differences between patients with and without ARDS were screened by univariate analysis. Then, Support Vector Machine (SVM), Ensembles of Decision Trees (EDTs), Bayesian Classifier (BC), and nomogram models were constructed and optimized after feature screening based on these parameters. Five-fold cross-validation was used to train each model. A test set was used to evaluate the predictive performance of the four models.

**Results:**

A total of 83 (18.04%) of 460 patients with AP developed ARDS. Thirty-one features with significant differences between the groups with and without ARDS in the training set were used for modeling. The Partial Pressure of Oxygen (PaO_2_), C-reactive protein, procalcitonin, lactic acid, Ca^2+^, the neutrophil:lymphocyte ratio, white blood cell count, and amylase were identified as the optimal subset of features. The BC algorithm had the best predictive performance with the highest AUC value (0.891) than SVM (0.870), EDTs (0.813), and the nomogram (0.874) in the test set. The EDT algorithm achieved the highest accuracy (0.891), precision (0.800), and F1 score (0.615), but the lowest FDR (0.200) and the second-highest NPV (0.902).

**Conclusions:**

A predictive model of ARDS complicated by AP was successfully developed based on ML. Predictive performance was evaluated by a test set, for which BC showed superior predictive performance and EDTs could be a more promising prediction tool for larger samples.

## Highlights

• ML can be a practical and effective early prediction method of AP complicated by ARDS.
• PaO_2_, CRP, PCT, LA, Ca^2+^, NLR, WBC, and AMY were used as the optimal subset of features to early identify AP patients with a high risk for developing ARDS in ML.
• BC was the superior predictive model and EDTs could be promising for predicting large samples.

## Introduction

Acute Pancreatitis (AP) is a common inflammatory disorder that can lead to Systemic Inflammatory Response Syndrome (SIRS), local and systemic complications, and life-threatening organ injury or Multiple Organ Failure (MOF). Although most patients (80%) develop a mild episode of AP with a good prognosis, about 20% develop moderately severe or severe AP (MSAP or SAP) with local complications and transient or persistent organ failure.^1^

Acute Respiratory Distress Syndrome (ARDS) is a syndrome of inflammatory pulmonary edema that causes hypoxia and is associated with increased permeability of the lung epithelium^2^ and vascular endothelium that occurs in approximately 30% of patients with SAP.^3^ The lung is often damaged initially during AP, and ARDS is a common complication. Respiratory failure is the most common type of organ failure (92%) during the early and late phases of AP with a 37% mortality rate.^4^ The main cause of the high fatality rate may be related to the lack of predicting early organ failure and the management strategy. However, ARDS is somewhat preventable, and clinical outcomes may improve after appropriate interventions during the early phase of ARDS.^5^ Therefore, it is important to identify patients with AP early who are at high risk for developing ARDS. A more accurate and convenient early predictive tool is needed to help physicians identify and prevent progression to ARDS.

Applications of Artificial Intelligence (AI), such as Machine Learning (ML), have become more practical in the field of disease outcome prediction with continuous improvements in computer science. ML is an emerging field and has widely infiltrated clinical medical studies. Notably, ML analysis relies on different deep iterative algorithms to integrate candidate variables, so highly accurate predictions can be obtained.

This study developed ARDS risk prediction models for patients with AP in the early stage from a larger set of clinical parameters. All of the models were tested in an independent cohort of AP patients. The ability to accurately risk stratifies may facilitate more timely interventions that are conducive to high-risk ARDS management via early identification.

## Methods

### Participants

The authors performed a retrospective observational study of AP patients based on the STROBE checklist. Our cases were from patients who were admitted to the Xuanwu Hospital of Capital Medical University from January 2017 to August 2022. The hospital has an independent acute pancreatitis therapy center, including a gastroenterology intensive care unit. The inclusion criteria were age ≥18 years and a confirmed diagnosis of AP. The exclusion criteria were more than 24h after onset of symptoms, history of AP attacks, AP with chronic obstructive pulmonary disease, AP with malignant tumors, AP with chronic heart failure or kidney disease, AP and pregnant, or AP with HIV/AIDS or another immune-deficiency disorder. All patients received standard medical treatment to manage AP according to international guidelines.

The AP diagnostic criteria were set up according to the revised Atlanta classification of acute pancreatitis 2012.^6^ At least two of the following three criteria had to be satisfied for the AP diagnosis: abdominal pain, increased serum levels of Amylase (AMY) and/or Lipase (LPS) to at least three times the normal upper limit, and image findings of AP in abdominal ultrasonic and/or a Computed Tomography (CT) scan. Hypertriglyceridemia associated with AP was defined as levels of triglycerides ≥ 11.3 mmoL/L (1000 mg/dL) or ≥5.65 mmoL/L (500 mg/dL) accompanied by milky serum.^6^

The ARDS diagnosis was made according to the Berlin definition as acute hypoxemia, a decrease in the PaO_2_/FiO_2_ index <300 mmHg, and bilateral lung infiltration in an X-Ray/CT scan that was not totally illuminated by fluid overload or cardiac failure.^7^ Arterial blood gas analysis was performed for patients as well as when a patient developed dyspnea during hospitalization.

### Data collection

The data included clinical characteristics and laboratory findings, and patients were admitted in ≤24h. Demographic and clinical features, including age, gender, Body Mass Index (BMI), etiology (hypertriglyceridemia, biliary, alcohol, and other), Heart Rate (HR), Respiratory Rate (RR), body Temperature (T), and history of hypertension, diabetes, and Non-Alcoholic Fatty Liver Disease (NAFLD) were recorded. The 42 laboratory parameters obtained at admission are shown in Table 1.

**Table 1:** Baseline characteristics of AP patients with or without ARDS.

Characteristics	Non-ARDS (n = 377)	ARDS (n = 83)	Total (n = 460)	p*-*value
**Demographics**				
Male, n (%)	273 (72.41%)	57 (68.67%)	330 (71.74%)	0.493
Age, year	42.00 (35.00, 59.00)	45.00 (34.00, 60.00)	43.50 (35.00, 59.00)	0.537
BMI, kg/m^2^	26.44 (24.22, 29.58)	28.68 (24.96, 32.14)	26.67 (24.29, 30.12)	0.002
Hypertension, n (%)	137 (36.34%)	37 (44.58%)	174 (37.83%)	0.161
Diabetes mellitus, n (%)	162 (42.97%)	42 (50.60%)	204 (44.35%)	0.205
NAFLD, n (%)	234 (62.07%)	55 (66.27%)	289 (62.83%)	0.474
Etiology, n (%)				0.003
Hypertriglyceridemia	156 (41.38%)	52 (62.65%)	208 (45.22%)	<0.001
Biliary	69 (18.30%)	14 (16.87%)	83 (18.04%)	0.758
Alcoholic	43 (11.41%)	4 (4.82%)	47 (10.22%)	0.073
Other	109 (28.91%)	13 (15.66%)	122 (26.52%)	**0.013**
**Clinical signs**				
HR, beats/min	80.00 (70.00, 94.00)	100.00 (80.00, 116.00)	82.00 (72.00, 98.00)	**<0.001**
RR, breaths/min	19.00 (17.00, 21.00)	24.00 (20.00, 27.00)	20.00 (17.00, 23.00)	**<0.001**
Temperature, Celsius	36.50 (36.20, 36.90)	36.80 (36.40, 37.40)	36.60 (36.20, 37.00)	**<0.001**
**Routine blood test**				
WBC, ×10^9^/L	9.74 (7.40, 12.28)	12.27 (9.58, 16.41)	10.25 (7.92, 13.22)	<0.001
NEUT, ×10^9^/L	7.78 (5.49, 10.44)	10.63 (8.32, 14.48)	8.20 (6.08, 11.09)	<0.001
LYM, ×10^9^/L	1.28 (0.95, 1.67)	1.05 (0.76, 1.28)	1.24 (0.91, 1.62)	<0.001
NLR	5.90 (3.93, 8.99)	11.02 (7.21, 15.88)	5.55 (4.32, 10.29)	**<0.001**
HCT, %	41.80 (38.50, 44.80)	43.40 (39.90, 46.80)	41.90 (38.60, 45.30)	0.005
PLT, ×109/L	218.00 (179.00, 262.00)	217.00 (165.00, 269.00)	218.00 (176.25, 264.75)	0.737
RDW, %	12.90 (12.40, 13.40)	13.00 (12.70, 13.60)	12.90 (12.40, 13.40)	0.054
MPV, fl	10.30 (9.80, 11.00)	10.70 (10.00, 11.30)	10.40 (9.80, 11.00)	0.003
Biochemical test				
TB, µmoL/L	15.23 (11.37, 20.82)	15.27 (11.43, 21.10)	15.26 (11.38, 20.82)	0.963
DB, µmoL/L	4.53 (2.89, 6.97)	4.72 (2.73, 6.22)	4.55 (2.86, 6.93)	0.605
ALB, g/L	39.44 ± 4.35	36.72 ± 6.08	38.95 ± 4.82	<0.001
AGR	1.38 ± 0.31	1.23 ± 0.34	1.35 ± 0.32	<0.001
ALT, IU/L	23.00 (15.00, 38.00)	22.00 (15.00, 34.00)	22.00 (15.00, 38.00)	0.969
AST, IU/L	24.00 (19.00, 32.00)	28.00 (20.00, 44.00)	24.00 (19.00, 33.25)	0.019
LDH, IU/L	201.00 (170.25, 243.75)	311.50 (222.75, 433.75)	209.50 (174.25, 264.00)	<0.001
GGT, IU/L	43.00 (22.00, 82.50)	52.00 (31.00, 97.00)	44.00 (23.00, 85.00)	0.113
ALP, IU/L	70.00 (57.00, 84.50)	64.00 (53.00, 82.00)	69.00 (56.00, 84.00)	0.108
BUN, mmoL/L	4.10 (3.25, 5.23)	4.83 (3.53, 6.27)	4.21 (3.30, 5.38)	0.003
Cr, µmoL/L	60.00 (50.00, 70.00)	60.00 (49.00, 74.00)	60.00 (50.00, 70.00)	0.735
GLU, mmoL/L	7.31 (5.76, 11.25)	10.03 (7.42, 13.68)	7.90 (6.07, 11.93)	**<0.001**
TG, mmoL/L	2.17 (0.93, 6.75)	5.34 (1.30, 20.61)	2.40 (0.98, 8.49)	**<0.001**
Ca^2+^, mmoL/L	2.17 (2.08, 2.26)	2.07 (1.86, 2.21)	2.16 (2.05, 2.26)	**<0.001**
K+, mmoL/L	3.92 (3.70, 4.15)	3.93 (3.64, 4.25)	3.92 (3.70, 4.16)	0.929
AMY, IU/L	151.00 (70.00, 419.00)	293.00 (144.00, 630.00)	176.00 (72.00, 444.00)	**<0.001**
LPS, U/L	179.10 (84.15, 463.83)	378.00 (150.00, 684.95)	216.90 (89.00, 517.55)	**<0.001**
**Coagulogram**				
PT, seconds	13.40 (12.90, 14.00)	13.75 (13.10, 14.43)	13.50 (12.90, 14.03)	**0.018**
TT, seconds	15.20 (14.60, 16.00)	15.10 (14.40, 15.85)	15.20 (14.50, 15.90)	0.436
APTT, seconds	37.10 (33.80, 40.60)	37.95 (34.15, 40.90)	37.30 (33.80, 40.70)	0.578
INR	1.03 (0.98, 1.08)	1.05 (1.00, 1.12)	1.03 (0.98, 1.09)	0.059
FIB, g/L	4.59 (3.47, 5.95)	5.63 (4.10, 7.56)	4.66 (3.53, 6.21)	**<0.001**
Dimer, µg/mL	0.91 (0.43, 1.95)	1.50 (0.64, 3.24)	0.99 (0.46, 2.16)	**<0.001**
**Inflammatory markers**				
CRP, mg/L	72.50 (18.40, 132.00)	238.00 (73.00, 348.00)	79.25 (21.65, 172.50)	**<0.001**
IL-6, pg/mL	23.99 (10.29, 63.05)	106.10 (58.60, 238.23)	31.36 (11.61, 84.27)	**<0.001**
PCT, ng/mL	0.07 (0.04, 0.18)	0.38 (0.15, 0.97)	0.09 (0.05, 0.26)	**<0.001**
**Arterial blood gases**				
PaO_2_, mmHg	79.10 (72.83, 87.00)	64.20 (60.70, 72.00)	77.10 (69.90, 85.90)	**<0.001**
PaCO_2_, mmHg	38.60 (35.63, 41.20)	36.80 (33.30, 40.90)	38.30 (35.40, 41.10)	**0.014**
PaO_2_: FiO_2_	376.67 (346.79, 414.29)	305.71 (289.05, 342.86)	367.14 (332.86, 409.05)	**<0.001**
SaO_2_, %	95.80 (94.50, 96.90)	92.50 (90.80, 94.90)	95.50 (93.80, 96.60)	**<0.001**
LA, mmoL/L	1.50 (1.20, 2.00)	2.00 (1.50, 2.90)	1.60 (1.20, 2.10)	**<0.001**

BMI, Body Mass Index; NAFLD, Non-Alcoholic Fatty Liver Disease; HR, Heart Rate; RR, Respiratory Rate; WBC, White Blood Cell; NEUT, Neutrophil; LYM, Lymphocyte; NLR, Neutrophil-Lymphocyte Ratio; HCT, Hematocrit; PLT, Platelet; RDW, Red blood cell Distribution Width; PDW, Platelet Distribution Width; MPV, Mean Platelet Volume; TB, Total Bilirubin; DB, Direct Bilirubin; ALB, Albumin; AGR, Albumin-Globulin Ratio; ALT, Alanine Aminotransferase; AST, Aspartate Transaminase; LDH, Lactic Dehydrogenase; GGT, γ-Glutamyltransferase; ALP, Alkaline Phosphatase; BUN, Blood Urea Nitrogen; Cr, Creatinine; GLU, Glucose; TG, Triglyceride; Ca^2+^, Calcium ion; K^+^, Potassium ion; AMY, Amylase; LPS, Lipase; PT, Prothrombin time; TT, Thrombin Time; APTT, Activated Partial Thromboplastin Time; INR, International Normalized Rratio; FIB, Fibrinogen; CRP, C-Reactive Protein; IL-6, Interleukin-6; PCT, Procalcitonin; PaO_2_, Partial Pressure of Oxygen; PaCO_2_, Partial Pressure of Carbon Dioxide; PaO_2_: FiO_2_, Partial Pressure of Oxygen/Fraction of inspiration Oxygen; SaO_2_, Arterial Oxygen Saturation; LA, Lactic Acid.

### Statistics

Continuous variables are presented as a median and interquartile range for skewed distributions or as mean ± standard deviation for the variables with a normal distribution, while categorical variables are presented as frequencies and proportions. Student's *t*-test or the nonparametric Mann-Whitney test was applied to compare the ARDS and non-ARDS groups. Pearson's Chi-Square or Fisher's exact test was used for the categorical data. Statistical analyses were performed using SPSS 23.0 software (SPSS Inc., Chicago, IL, USA). A two-sided p-value < 0.05 was considered significant.

### Development of the ML models

The missing values in the original data were multiple interpolated using the bagImpute method based on the bagged tree model. The complete data were randomly distributed into the training and testing cohorts at a 4:1 ratio. The training cohort was applied to develop the model with ML algorithms, and variables were inputted that had significant differences (p<0.05) in the univariate analysis between AP patients with or without ARDS to predict the risk for ARDS. Four ML algorithms were selected, including Support Vector Machine (SVM), Ensembles of Decision Trees (EDTs), Bayesian Classifier (BC), and the nomogram algorithm. These algorithms were applied using Matlab 2014 (MathWorks, Natick, MA, USA). Internal validation was accomplished with five-fold cross-validation of the training set in each ML model after selecting the optimal feature subset. Because five-fold was used for the validation set, the above process was repeated 10 times.

### Evaluation and testing of the ML models

The final Receiver Operating Characteristics (ROC) curve, the average Area Under the Curve (AUC), accuracy, precision, recall, True Negative Rate (TNR), F1 score, Negative Predictive Value (NPV), and False Discovery Rate (FDR) was utilized to evaluate and compare the predictive performance of the models. The four ML models trained on the optimal feature subsets were tested with an independent test set.

## Results

### Baseline demographic and clinical characteristics

**Fig. 1:**
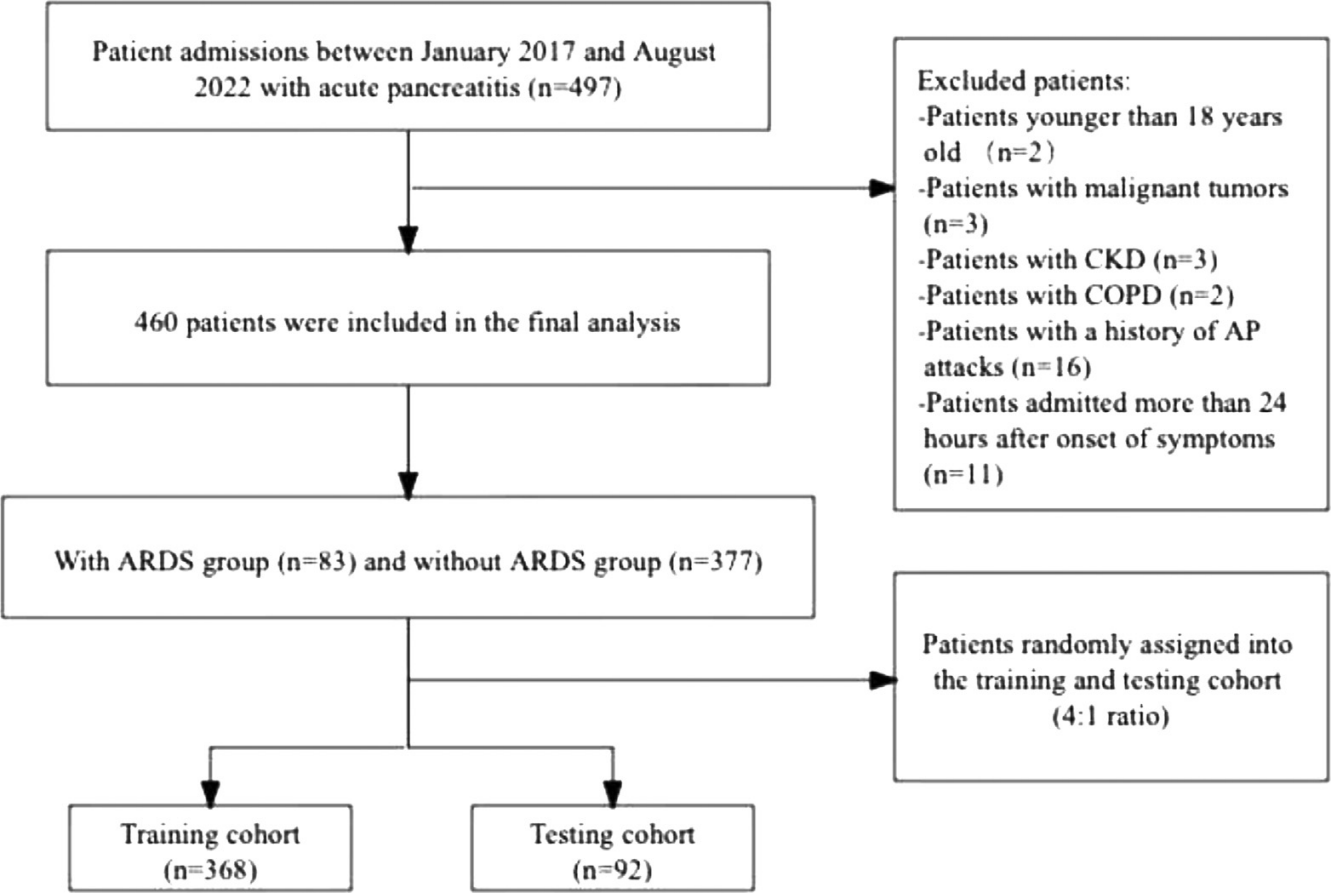
Flow diagram of patient enrollment and cohort selection.

In all, 497 patients with AP were initially identified and 37 were excluded according to the exclusion criteria. Ultimately, 460 patients were included in the study (Fig. 1). The characteristics of the patients with and without ARDS are summarized in Table 1. ARDS occurred in 83 of the 460 patients (18.04%). In all, 368 patients were included in the training cohort and 92 in the testing cohort. ARDS occurred in 66 patients (17.93%) in the training cohort and 17 (18.48%) in the testing cohort. Hypertriglyceridemia (45.22%) was the most common cause of AP.

Thirty-one parameters differed significantly between patients with and without ARDS (Table 1). A significant difference was observed in the etiology of hypertriglyceridemia between the two groups. No differences in gender, age, or history of hypertension, diabetes, or NAFLD were observed between the two groups.

### Feature selection and development of the ML models

**Fig. 2:**
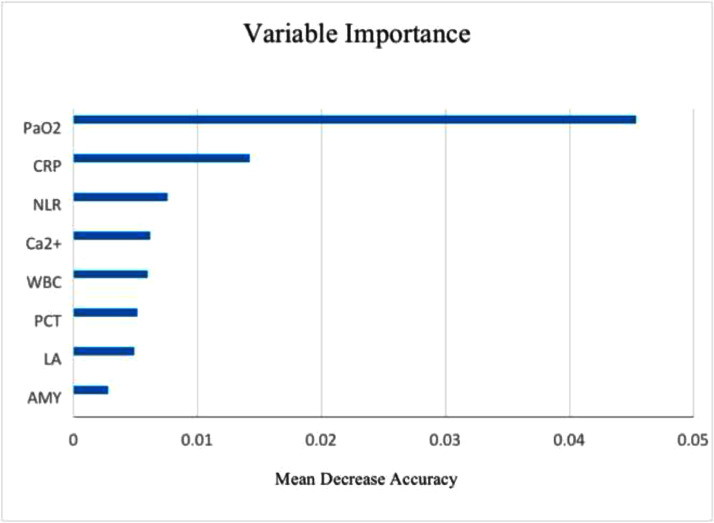
Variable importance in the optimal feature subset, showing that PaO_2_ was the most important feature, followed by CRP, NLR, Ca^2+^, WBC, PCT, LA, and AMY. Abbreviations: PaO_2_, Partial Pressure of Oxygen; CRP, C-Reactive Protein; NLR, Neutrophil-Lymphocyte Ratio; Ca^2+^, Calcium Ion; WBC, White Blood Cell Count; PCT, Procalcitonin; LA, Lactic Acid; AMY, Amylase.

The features that were significantly different between the two groups were used for feature selection using the random forest algorithm and the Recursive Feature Elimination (RFE) strategy to determine an optimal subset of features that effectively predicted the risk for ARDS in patients with AP (Supplementary Fig. 1). As some features had strong internal correlations, the authors tested all feature correlations and retained the features with the strongest correlations using the target variable ARDS (Supplementary Fig. 2). Ultimately, the best eight features (Fig. 2) were identified as the optimal subset of features. These were entered into the ML models. To build a probabilistic model of the objective function and to select the most promising set of hyperparameters to evaluate, the authors optimized the ML models using a Bayesian hyperparameter optimizer (Supplementary Fig. 3).

### Feature importance in the optimal feature subset

The authors quantified the importance of each feature in the optimal feature subset using an RFE strategy in the random forest algorithm. As shown in Fig. 2, PaO_2_ was the most important feature, followed by CRP, NLR, Ca^2+^, WBC, PCT, LA, and AMY in order of importance in predictiveness.

### ML model training and validation

**Fig. 3:**
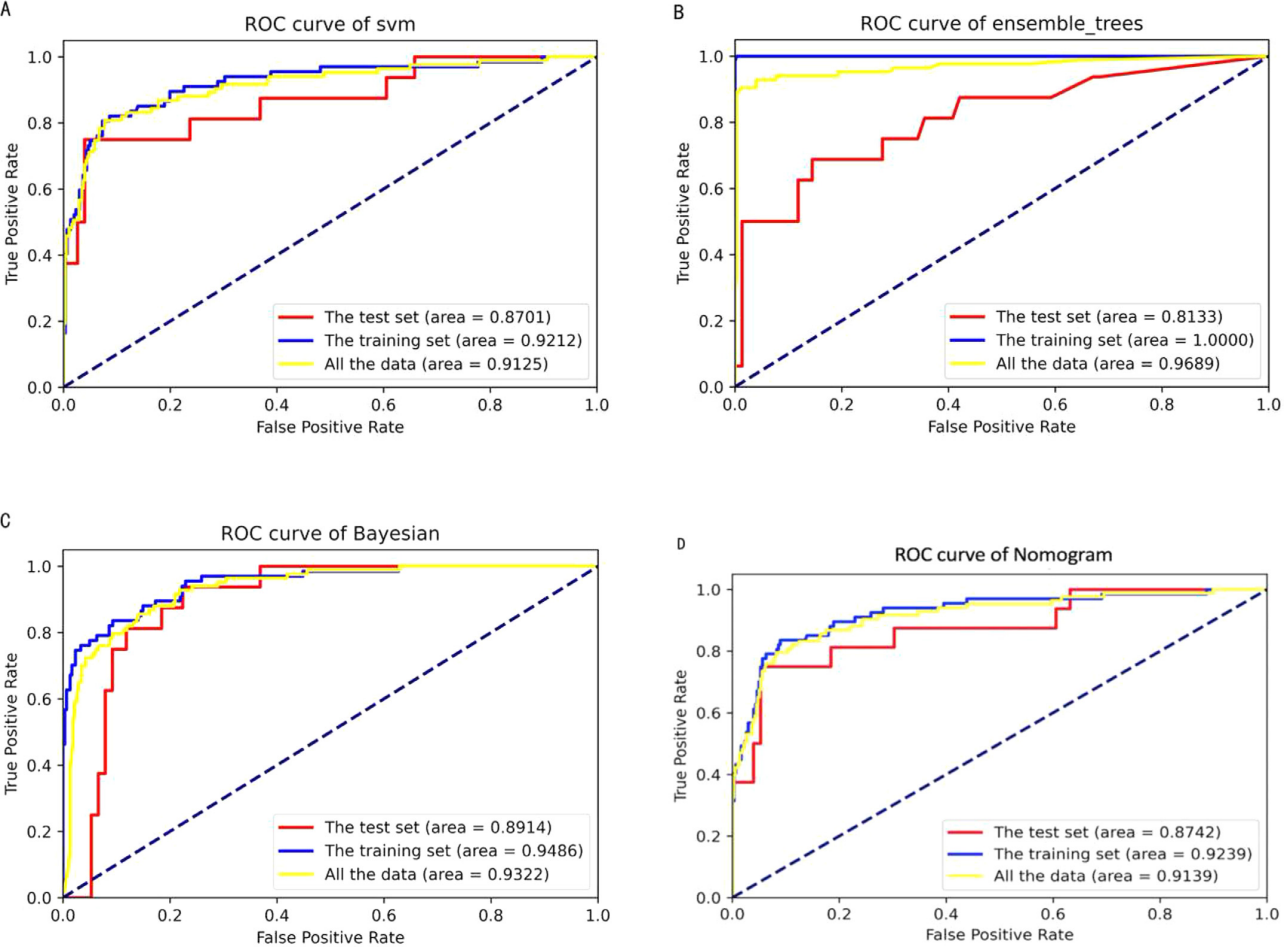
The ROC curves of different models in the training set, test set and all data. (A) SVM. (B) EDTs. (C) BC. (D) Nomogram.

**Fig. 4:**
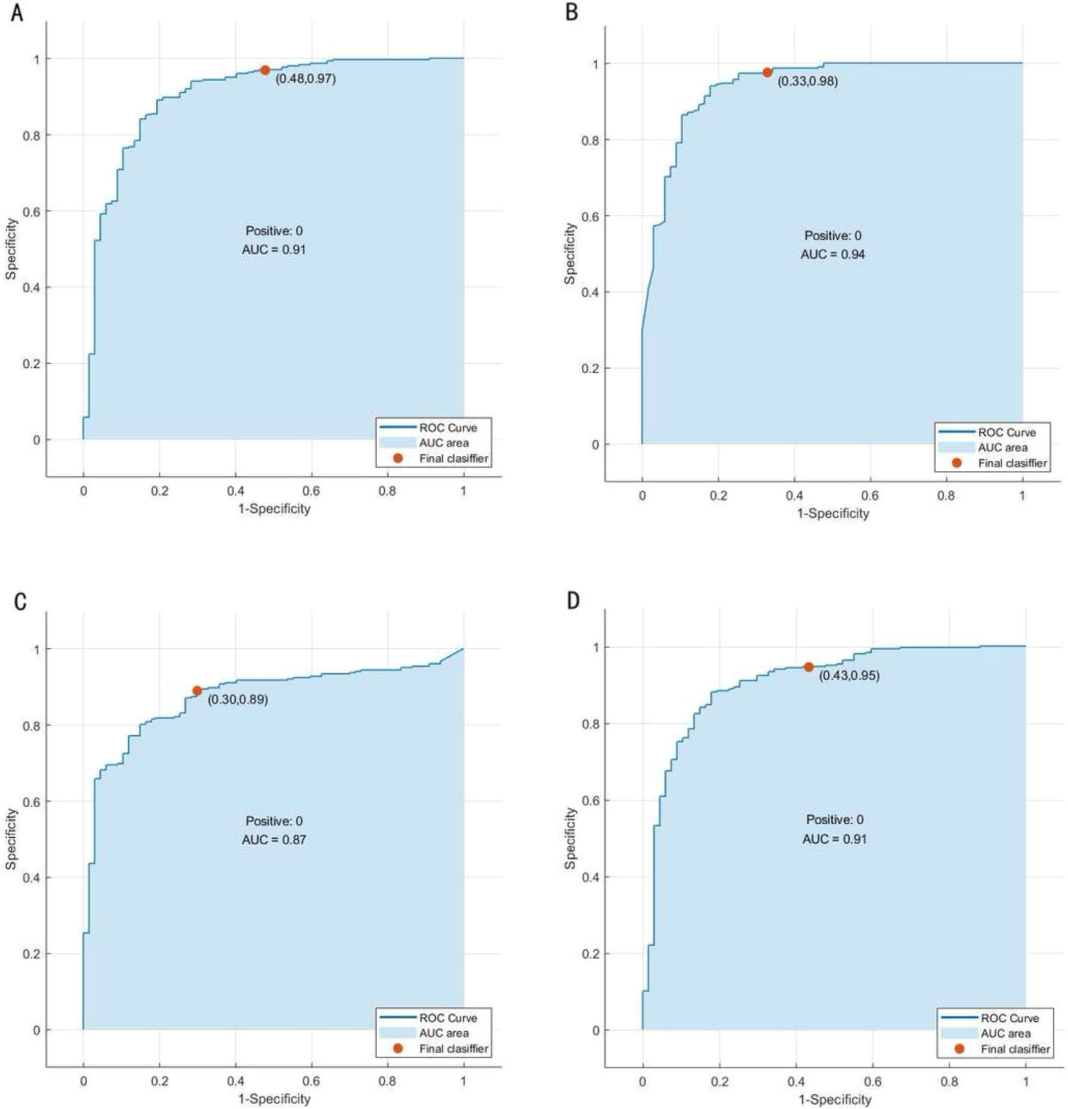
The ROC curves of different models after five-fold cross-validation of the training set. (A) SVM. (B) EDTs. (C) BC. (D) Nomogram.

**Fig. 5:**
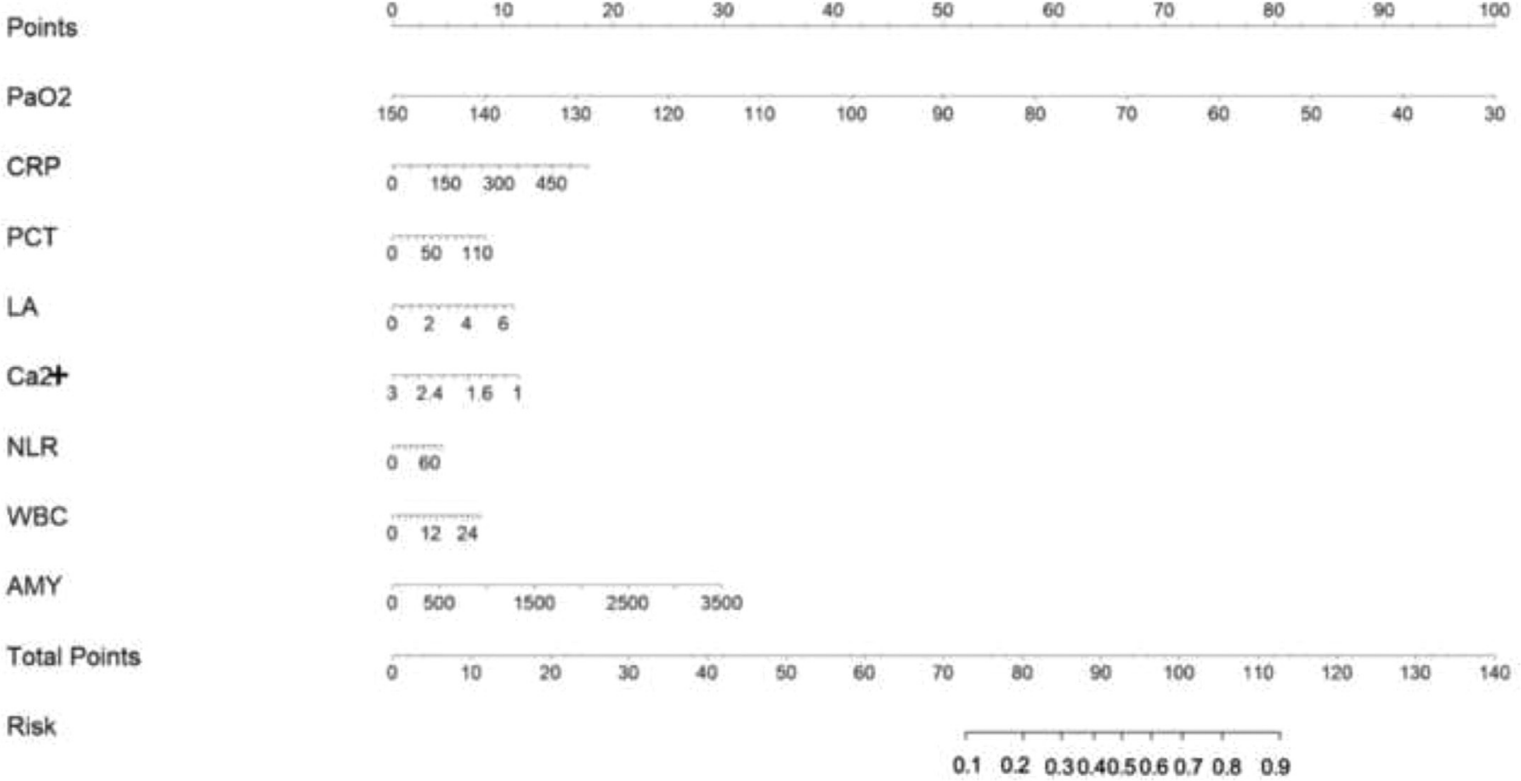
Early ARDS prediction nomogram in patients with AP.

The ROC curves of the four different models for predicting ARDS are shown in Fig. 3. Fig. 4 shows the ROC curves of the models after the five-fold cross-validation of the training set. The AUC values of the optimal feature subset in the SVM, EDT, BC, and nomogram models were 0.91, 0.94, 0.87, and 0.91, respectively. The EDT algorithm achieved the highest AUC, accuracy, precision, recall, TNR, F1 score, and NPV compared to the other three algorithms. Table 2 presents a set of detailed metrics for the four models in the training dataset. Fig. 5 is a nomogram of the visual results of logistic regression, indicating the association between the predictor variables and the occurrence of ARDS in patients with AP.

**Table 2:** Evaluation metrics of different models in training set.

	AUC	Accuracy	Precision	Recall	TNR	F1 Score	NPV	FDR
**SVM**	0.912	0.894	0.804	0.552	0.970	0.655	0.907	0.196
**EDTs**	0.940	0.997	1.00	0.985	1.00	0.992	0.997	0.00
**BC**	0.873	0.918	0.785	0.761	0.953	0.773	0.947	0.215
**Nomogram**	0.912	0.891	0.765	0.582	0.960	0.661	0.912	0.235

### Comparison of predictive performance among the four models

The authors generated four models to predict the early onset of ARDS in AP patients after admission. Then, the authors evaluated the predictive performance of each ML model trained using the optimal feature subset. All detailed performance metrics obtained by the four models in the testing set are shown in Table 3. The AUC values were 0.870 for SVM, 0.813 for EDTs, 0.891 for BC, and 0.874 for the nomogram. The ROC curve obtained for each model in the testing set is shown in Fig. 3. The AUC value demonstrated that the BC model achieved the best predictive effect with the highest AUC of 0.891, recall of 0.563, and NPV of 0.909 compared with other models. EDTs achieved good predictive performance with the highest accuracy (0.891), precision (0.800), and F1 score (0.615), but the lowest FDR (0.200) and the second-highest NPV (0.902).

**Table 3:** Predictive performance of different models in testing set.

	AUC	Accuracy	Precision	Recall	TNR	F1 Score	NPV	FDR
**SVM**	0.870	0.870	0.750	0.375	0.974	0.500	0.881	0.250
**EDTs**	0.813	0.891	0.800	0.500	0.974	0.615	0.902	0.200
**BC**	0.891	0.859	0.600	0.563	0.921	0.581	0.909	0.400
**Nomogram**	0.874	0.870	0.750	0.375	0.974	0.500	0.881	0.250

## Discussion

ARDS is the triggering point in the development of MOF in patients with AP, which is associated with high mortality.^8^ Therefore, it is extremely important to predict the risk for ARDS early, which can help prevent the development of ARDS and further deterioration of other organs. However, there are no validated serum biomarkers or scoring systems to predict ARDS in patients with AP. ML techniques are increasingly recognized by medical professionals because of their extraordinary ability to analyze information. Here, the authors developed and tested four ML algorithms as convenient tools to predict ARDS complicated by AP in the early phase. The authors performed correlation analysis on the features and quantified the importance of each feature on the target variable. A set of high-quality optimal features was obtained, and the prediction models were constructed with the least number of features and the lowest redundancy of feature information; hyperparameter optimization was performed for each model.

Clinical data from a routine blood test, biochemistry, coagulogram, inflammatory markers, and arterial blood gas analysis were collected to develop the ML models. Although the four models all yielded satisfactory predictive performance, the BC and EDTs models more accurately predicted the risk for ARDS in patients with AP. BC had the best predictive performance using the testing set. EDTs had the highest AUC value and superior accuracy, specificity, and sensitivity in the training set.

In this study, a lower PaO_2_ and a lower Ca^2+^ level, as well as a higher CRP, PCT, LA, NLR, WBC, and AMY at admission were correlated with a higher risk of developing ARDS in patients with AP. Among them, PaO_2_ was the foremost feature.

Hypoxemia is not only a diagnostic criterion for ARDS, but the respiratory symptoms it causes are the earliest clinical manifestations of AP.^9^ As no specific drug treatment exists for ARDS, good supportive care reduces damage and improves the prognosis.^10^^,^^11^ Therefore, early diagnosis benefits patients. In this study, the arterial PaO_2_ in patients with ARDS was 64.20 (60.70, 72.00) mmHg, which was significantly lower than that of patients without ARDS with 79.10 (72.83, 87.00) mmHg, suggesting that ARDS should be suspected in all AP patients once hypoxemia and related symptoms appear.^11^

CRP was the second-most important feature for predicting ARDS in our study and has been used to predict the severity of AP. This result also confirms that inflammation is closely associated with ARDS in patients with AP, which is consistent with the prevailing view that systemic inflammatory response syndrome is the first stage of ARDS in AP patients.^12^^,^^13^ The WBC count and NLR had early predictive value for the severity of AP and persistent organ failure,^14^, ^15^, ^16^ and are also clinical markers for predicting mortality and fatal complications in patients with ARDS.^17^^,^^18^. The NLR served as the third-most important predictive feature in our models, similar to a previous study.^19^ PCT is associated with MOF and ARDS in patients with SAP.^20^, ^21^, ^22^ The authors observed that patients with a higher PCT at admission were more likely to develop ARDS, consistent with previous findings.^23^ The significantly lower Ca^2+^ concentrations in patients with ARDS compared to those without ARDS suggests that tissue necrosis triggers a systemic inflammatory response, resulting in the release of inflammatory cells and mediators, which further triggers ARDS. Here, LA and Ca^2+^ were the independent variables in ARDS, indicating that these features should be monitored. Although serum levels of AMY were not associated with AP severity, AMY levels at admission were a risk factor for predicting ARDS, similar to previous results.^24^ However, further study on the relationship between these factors in patients with AP is warranted.

Hypertriglyceridemia-induced AP (HTG-AP) varies from 10% to 30% in different countries^25^, ^26^, ^27^ and high TG levels are associated with the severity and clinical prognosis of AP.^28^^,^ ^29^ HTG-AP is increasing gradually, especially in China.^30^, ^31^, ^32^, ^33^ In our study, hypertriglyceridemia accounted for 45.22% of the etiology, consistent with the 40%–49% reported in recent studies.^19^^,^^23^. In addition, the authors found that the proportion of HTG-AP was significantly higher in the ARDS group than in the group without ARDS, consistent with the results of pneumonia-initiated ARDS.^34^ This result may be due to the fat embolism syndrome caused by high levels of free fatty acids in HTG-AP patients, which can lead to pulmonary vascular endothelial damage and microcirculatory disorder. No significant differences in age or comorbidities such as diabetes, hypertension, or NAFLD were detected between the two groups, suggesting that, unlike pneumonia-initiated ARDS, age and comorbidities cannot be used as predictors of ARDS caused by AP.

Two recent studies used nomograms to predict ARDS in AP with AUC values of 0.821 and 0.814, respectively.^19^^,^^23^ The authors performed a two-step feature selection strategy to filter the optimal subset of features, followed by optimizing the parameters to develop the predictive models. Compared to complex scoring systems (e.g., APACHE II), ML models are convenient to determine prediction probability. ML has the advantage of analyzing the nonlinear relationships between various markers and ARDS over traditional statistical methods, which allows for early prediction before significant changes in classical metrics occur. Based on the prediction performance, the authors recommend the BC algorithm with the highest AUC value of 0.891, indicating that it is more robust in extrapolation. Second, the authors recommend the EDT algorithm with superior evaluation metrics from the training set, indicating its strongest fitting ability. The unbalanced distribution of the original data may have directly affected the extrapolation ability of the model. Therefore, the authors believe that BC provided the most accurate prediction given the available data and that EDTs have greater potential as sample size increases.

Several limitations of our study should be mentioned. First, our data were derived from a single AP center and the number of cases was small. Some differences in the performance of the ML models may occur when applied to datasets from different institutions with different distributions of covariates. Second, the authors reported ARDS as a dichotomous variable (presence or absence) rather than across time; thus, our results cannot predict the development of ARDS. Third, the small sample size prevented the evaluation of subgroups according to ARDS severity. Finally, our study was retrospective and there may be patient selection bias, which is an unavoidable limitation of such studies. Further multicenter prospective studies with larger samples should be conducted to verify our ARDS predictive models in patients with AP.

## Conclusions

The authors developed and validated four models to predict ARDS early in patients with AP based on the SVM, BC, EDTs, and a nomogram. PaO_2_, CRP, PCT, LA, Ca^2+^, the NLR, WBC, and AMY were the optimal subset of features. BC was the superior predictive model in the test set. Additionally, EDTs could be promising for predicting large samples.

### Abbreviations

ARDS, Acute Respiratory Distress Syndrome; AP, Acute Pancreatitis; MSAP, Moderately Severe Acute Pancreatitis; SAP, Severe Acute Pancreatitis; ML, Machine Learning; AI, Artificial Intelligence, SVM, Support Vector Machine; EDTs, Ensembles of Decision Trees; BC, Bayesian Classifier; SIRS, Systemic Inflammatory Response Syndrome; BMI, Body Mass Index; NAFLD, Non-Alcoholic Fatty Liver Disease; HR, Heart Rate; RR, Respiratory Rate; T, Body Temperature; ROC, Receiver Operating Characteristics; AUC, Area Under the Curve; TNR, True Negative Rate; NPV, Negative Predictive Value; FDR, False Discovery Rate.

## Conflicts of interest

The authors declare no conflicts of interest.

## Data Availability

The data presented in this study are available on request from the corresponding author.

## Appendix Supplementary materials

Image, application 1
