# Supplementary material for: Early prediction of acute respiratory distress syndrome complicated by acute pancreatitis based on four machine learning models
Source: Clinics (Sao Paulo). 2023 May 3;78:100215. doi: 10.1016/j.clinsp.2023.100215 (PMC10199163; doi:10.1016/j.clinsp.2023.100215)
Supplement: Supplementary file 1 [file mmc1.docx]

**CLINICS-D-23-00007_ Supplementary Material**

**Supplementary Figure 1** The optimal subset of features generated using Random Forest algorithm and Recursive Feature Elimination (RFE) strategy.


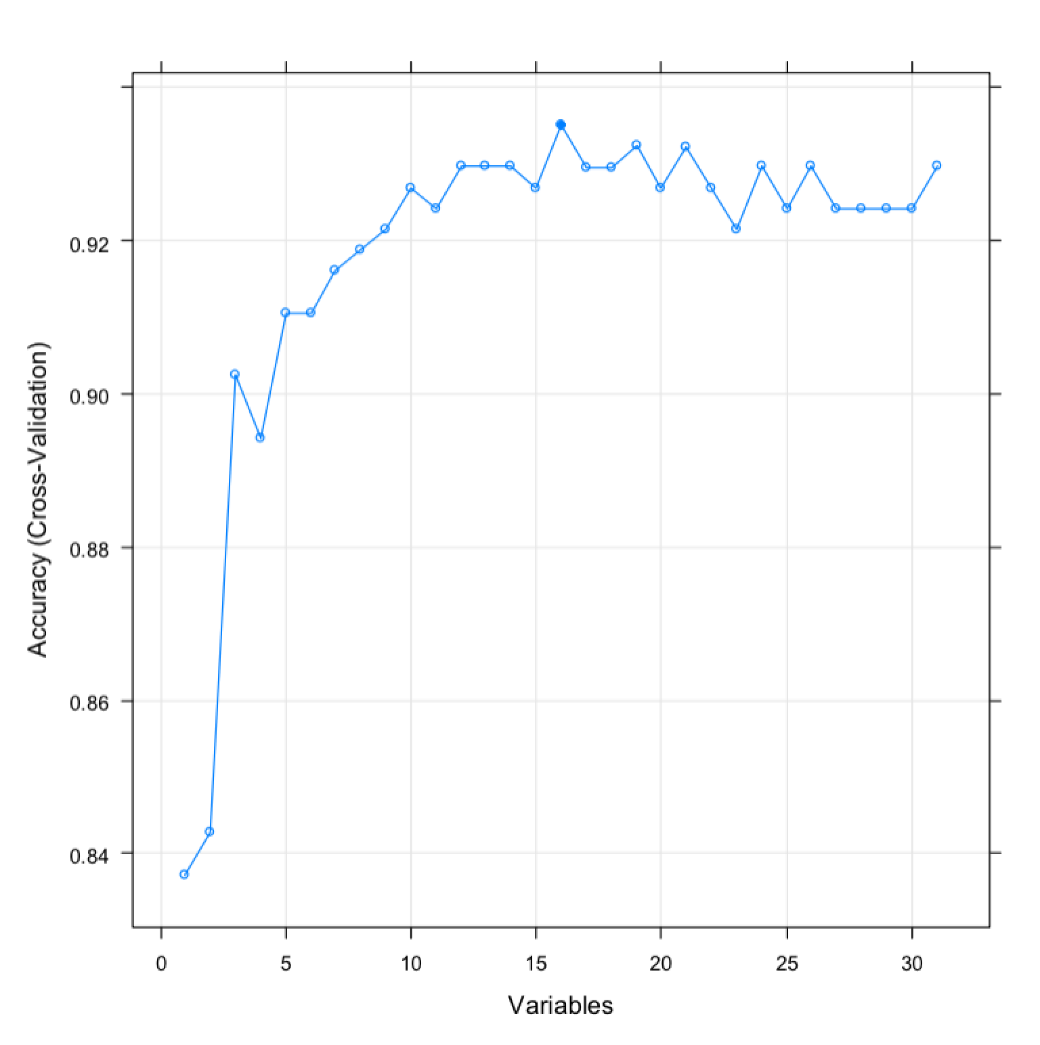


**Supplementary Figure 2** Correlations among different variables and between variables and ARDS.


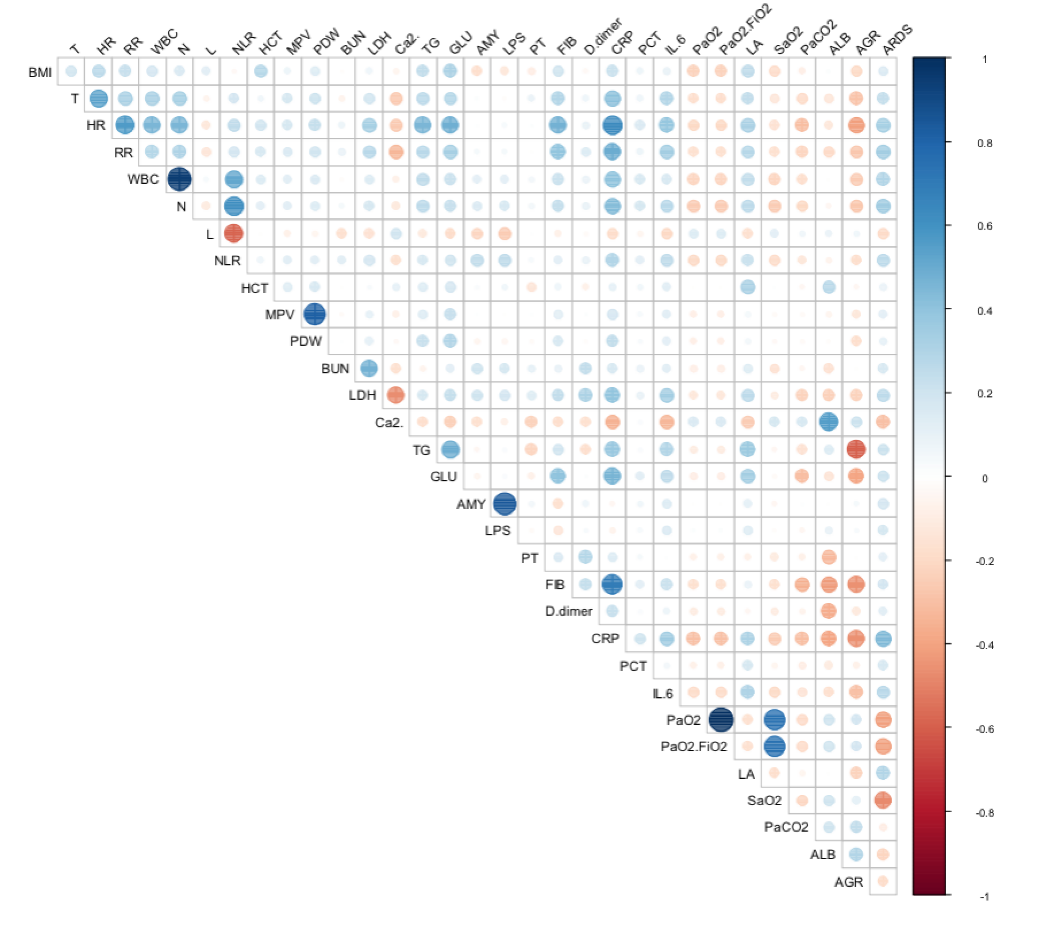


**Supplementary Figure 3** Hyperparameter optimization of different ML models. (A) SVM. (B) EDTs. (C) BC.
